# Chemical Lecture Notes

**Published:** 1890-12

**Authors:** 


CHEMICAL LECTURE NOTES. By H. M. Whelpley, M. D., Ph. G., F. R. M. S. Third edition. Published by the author at 2647 Olive Street, St. Louis, Mo. 1890. Price, $1.50.

This excellent little book is a complete summary of physics and chemistry for the use of students.

When a student attends lectures on chemistry and physics, he generally writes his own notes. These are necessarily imperfect, without arrangement, and of but little subsequent use. The small volume before us is a complete collection of these notes, clearly arranged and well printed. The student having this book in his hand needs no pencil and paper when attending lectures.

The professor who delivers the lectures need not tax himself by preparing any plan of lectures. He will find the whole thing in this book done for him, and more thoroughly, and above all more orderly.

The active practitioner, with little time at his command, who wishes to inform himself instantly upon some forgotten point without wading through a large mass of other material, will get what he wants from this book, which is a sort of miniature encyclopedia upon these subjects. W. M. J.

